# Review of true tales of medical physics: Insights into a life‐saving specialty, edited by Jacob Van Dyk

**DOI:** 10.1002/acm2.13944

**Published:** 2023-03-01

**Authors:** Robert Meiler

For most people the word “medical” conjures images of unusually attractive people in scrubs and lab coats, stethoscopes stuffed in oversized pockets, debating one or more life‐saving gambits. As the surgeon's and internist's eyes meet their Styrofoam coffee cups are not the only things that steam.

Instead, use the word “physics” and the images lean toward massive particle accelerators, glowing beams of energy, and colorful atoms dancing and spinning. But that is on a good day. Some poor souls have flashback memories of high school physics class, of the “mud on the wheel” problem, circuits, and too much math.

Somewhere between heaven and earth stands the medical physicist. To understand who these people are and their role in the use of radiation to image and heal, *True Tales of Medical Physics* presents an anthology of stories by a collection of high‐profile, “award‐winning” medical physicists. The 22 contributors to the book, though skewed toward North America, represent nine countries and include winners of the highest awards in the field of Medical Physics: Coolidge Award, Gold Medal…the list goes on. The short stories included in this work are organized into six categories and each is labeled with “More than…” to emphasize that, though each story has been included in a specific category, each touches on subjects from other categories as well. This degree of specificity may seem unusual, and it probably is, but it works.

In each of the 22 chapters an author takes the reader through events from their past that were either noteworthy or truly formative. From how each author entered this unheard‐of field, to “war stories” of their more colorful adventures, and to case studies with advice for those interested in the profession, each author's in‐depth account is engrossing and often quite funny. In each case, the author gives the reader not just the dry facts, but their feelings, fears, hopes, uncertainties, passions, etc. that led them, spurred them, and sustained them. Perhaps as valuable as their recounting of history are their reflections; what each learned from their histories.

*True Tales of Medical Physics* is unusual in that it aspires to be not only an anthology of memoirs, but something of a handbook that defines what a medical physicist is, not just for the lay person, but for practitioners of the discipline itself. It sets out to inform, guide, and inspire in a way not often seen in a book of this type. In the words of the editor, the contributors to this book were invited “to communicate what medical physics is and what medical physicists do to a broad audience including science students, graduate students and residents, experienced medical physicists and their family members, and the general public who are wondering about medical physics.”

Another example of the unusual approach taken by this book is the inclusion of a table in the prologue to summarize the relevance of each author's contribution to the various branches of medical physics. It is an approach one would take in writing a task group‐report for the American Association of Physicists in Medicine, North America's trade union for medical physicists. Perhaps old habits die hard, but the tables and the structure work very well in this book.

To give some context to the profession of medical physics, the first category of stories focuses upon the history of medical physics, where it came from, and how it arrived at its from in 2021.

“Medical Physics: More Than History” begins with a thoughtful essay on the definition of medical physics and a medical physicist. It explores the roots in early radiobiology that steered some physicists to focus upon clinical applications of radiation physics and away from the wider study of all areas of biophysics.

The blurred line between medical physics and biomedical engineering are touched upon for the first time and this is very much appreciated as it will be of great aid to those wishing to go into either field. The two have a great deal of overlap and deciding which degree to pursue and at which institution is not readily apparent.

An actually‐true parable about the unexpected performance of an inexpensive laundry cart that outperformed a costly freight elevator lightens the reading and helps illustrate some of the ways that technological innovation is not always predictable nor intuitively apparent. This theme is carried into the following two essays in this section which both present anecdotes that illustrate the circuitous path of progress both for the individuals themselves and the profession as a whole.

The next section presents three stories dealing with the clinical service aspect of a medical physicist's life. In this section the reader is given a tour of both a typical day‐in‐the‐life of a medical physicist as well as more insight into how the profession grew and the small coincidences and opportunities shaped each author's career. Included are more anecdotes about how the authors had to improvise and adapt to develop methods and technologies to meet the needs of their attending physicians.

The candor with which each authors tell their own tale breathes life into what could be a somewhat dry recounting of a job description. More than the tasks performed and the qualifications required, “Part II Medical Physics: More Than Clinical Service” helps the reader understand the person sitting in the office down the hall from the treatment suite, her or his focus, concerns, abilities, and drive.

Medical physics, though over 100 years old as a field of study, is still comparatively young in the family of sciences. There are still many questions to be answered and research is a vital part of this profession. “Part III Medical Physics: More than Research” devotes five stories and 120 pages to the topic. From the practical aspects of starting a career in medical physics research to examples and remembrances from people who were in the thick of some of the most significant developments in medical physics in the last 50 years. Both diagnostic imaging and radiotherapy physics are explored as well as the marriage of both in image‐guided radiotherapy.

The reader will find these glimpses into the personal thoughts of these researchers very interesting. Peeking into the workshop. Looking over the programmer's shoulder. Following each author through the process, from idea to dead‐end to inspiration to chance comment to another attempt to resolution is both engrossing and helpful examples for anyone starting a research career.

Foundational to the practice of medical physics, whether diagnostic imaging, nuclear medicine, radiotherapy, or the many industrial uses of radiation are the procedures for doing so safely. “Part IV Medical Physics: More than the Protection of the Public” includes three stories about the dangers posed to the general public by radioactivity.

Like most good science fiction movies, the danger begins when no one listens to the scientist. In the stories in this section the medical physicists had to wrestle with not just bureaucracy and neglect, but corruption and even civil war. The authors are unflinching in recounting the dangers both possible and imminent to the public and themselves. Agencies around the world maintain reports about radioactive incidents and near misses and many can be accessed online. But these personal experiences, the thoughts running through the heads of those on the ground, what they thought when they knew what they knew make these three stories some of the most gripping in this book.

“Part V Medical Physics: More than Teaching.” is not so much concerned with didactic lectures and training of medical physicists, (though each author recounts their experiences with their graduate students.) Instead, the stories in this section help the reader understand the ways in which a medical physicist interacts with both other professions and the public. As a niche and not‐widely known field, each medical physicist becomes something of an ambassador to her/his coworkers.

Though most people imagine physics research to be carried out in university laboratories surrounded by eager graduate students, in truth much of the groundbreaking work and translation to clinical application occurs in industry. The final section, “Part VI Medical Physics: More than Commercial Developments” includes four stories by authors who specifically pushed the boundaries of the technology and software available to medical physics and more generally, to radiology and radiation oncology. A reader with a specific interest in innovation and even commercialization within medical physics will find excellent advice in this section as well as great examples of how to pursue their goals.

There are wonderful insights and kernels of advice scattered throughout this book and anyone interested in a career in medical physics will find this book very informative. It provides valuable insight into the work and achievements of award‐winning physicists. At the same time, it would be an even more complete picture of the profession if it also included stories from the rank and file, the non‐academic, and the solo physicist. Their perspectives and experiences would round out this snapshot of the field of medical physics and add to a more complete resource for those wishing to pursue a career in clinical physics.

In seeking to answer the question of “what is a medical physicist?” *True Tales of Medical Physics* does a very good job of answering the unanswerable. Medical physics is a multi‐faceted profession and through the eyes of the authors of this book the reader is able to imagine a good outline of the profession, its opportunities, and what a day in the life for them is like.

For anyone who has wondered who invents the machines at the heart of modern medicine, or what happens behind the scenes before a CT appointment, this book introduces the reader to one of the invisible members of the care team. For anyone who likes both hard science and is interested in the clinical care of patients this book has many examples of people who have walked that path and can light the way. Lastly, for anyone who in interested in science and like a good yarn, *True Tales of Medical Physics* is as entertaining as it is informative.

The authors share their “tales” in a comfortable and accessible way that puts a very human face on their profession and creates a picture that any layperson will find identifiable. Readers may even wonder why there are not more medical dramas with medical physicists.

